# Exploring the social dimensions of AI integration in healthcare: a qualitative study of stakeholder views on challenges and opportunities

**DOI:** 10.1136/bmjopen-2024-096208

**Published:** 2025-06-27

**Authors:** Julia-Astrid Moldt, Teresa Festl-Wietek, Wolfgang Fuhl, Susanne Zabel, Manfred Claassen, Samuel Wagner, Kay Nieselt, Anne Herrmann-Werner

**Affiliations:** 1 TIME – Tübingen Institute for Medical Education, Faculty of Medicine, University of Tübingen, Tübingen, Germany; 2 Institute for Bioinformatics and Medical Informatics, University of Tübingen, Tübingen, Germany; 3 Department of Computer Science, University of Tübingen, Tübingen, Germany; 4 Department of Internal Medicine, University of Tübingen, Tübingen, Germany; 5 M3 Research Center, University Hospital Tübingen, Tübingen, Germany; 6 Deputy of the Rector & Sustainable Development, University of Tübingen, Tübingen, Germany; 7 Department of Internal Medicine VI/Psychosomatic Medicine and Psychotherapy, University Hospital Tübingen, Tübingen, Germany

**Keywords:** Artificial Intelligence, QUALITATIVE RESEARCH, MEDICAL EDUCATION & TRAINING, SOCIAL MEDICINE, PUBLIC HEALTH

## Abstract

**Abstract:**

**Objectives:**

This study aimed to investigate the opportunities and challenges associated with integrating artificial intelligence (AI) in healthcare by exploring the perspectives of various stakeholders. The objective was to provide a nuanced understanding of stakeholder views to address concerns and promote the acceptance and successful integration of AI technologies in medical practice.

**Design:**

This exploratory qualitative study used semi-structured interviews. Data were analysed using a combination of deductive and inductive coding, followed by content analysis to identify and develop categories.

**Setting:**

This study was conducted in Tübingen, Germany, within the framework of the TüKITZMed project (Tübingen AI Training Center for Medicine), between August 2022 and March 2023.

**Participants:**

A total of 38 stakeholders participated, including 6 lecturers, 9 clinicians, 10 healthcare students, 6 AI experts and 7 institutional stakeholders. Inclusion criteria included professionals involved in or affected by AI in healthcare, while exclusion criteria comprised individuals without relevant experience.

**Interventions:**

Not applicable.

**Outcome measures:**

The main outcome was the identification of thematic categories capturing stakeholders’ perceptions, expectations and concerns regarding the integration of AI in healthcare.

**Results:**

The analysis identified two main thematic categories: two main categories encompassing a total of 14 subcategories: (1) perceived opportunities of AI in medicine, including aspects of increased efficiency, reduced workload and improved patient safety and (2) perceived challenges of AI in medicine, such as its impact on medical decision-making and concerns about dependence on technology. These themes reflect diverse perspectives and insights across stakeholder groups.

**Conclusions:**

Diverse stakeholder perspectives offer valuable insights into the anticipated effects of AI in healthcare. Understanding these perspectives can support decision-makers in designing context-sensitive AI strategies and identifying areas for further professional and institutional development. Future research should monitor how these attitudes evolve in response to technological progress and real-world implementation.

**STRENGTHS AND LIMITATIONS OF THIS STUDY**

- The use of semistructured interviews provided flexibility, allowing participants to express their views in depth while ensuring that key topics were addressed.
- The iterative coding process facilitated the identification of recurring themes and nuanced insights from stakeholder perspectives.
- The small sample size and the participant demographics may limit the diversity of perspectives captured, potentially narrowing the contextual applicability of the findings to broader stakeholder views on artificial intelligence in healthcare.

## Introduction

 Artificial intelligence (AI) has the potential to transform various areas of medicine. AI systems are already able to precisely analyse complex medical images to detect diseases at an early stage and provide doctors with diagnostic support or improve workflows in the healthcare system. They can also help reduce medical errors and enable more individualised care—for example, through AI-supported monitoring in combination with telemedicine.^1 2^

Machine learning (ML), a core component of AI, enables systems to independently identify patterns and correlations in large datasets. Advanced ML techniques enhance pattern recognition in large data sets and facilitate the diagnosis and prediction of diseases in healthcare settings.^3 4^ AI algorithms are now widely used in diagnostic imaging to detect abnormalities, tumours, fractures and other medical conditions with high accuracy and speed.^5 6^

Moreover, ML-powered clinical decision support systems are already in use to help monitor patients with chronic or acute conditions in both remote settings and hospitals, including during postsurgical recovery and in intensive care units, using wireless body sensors.^7^ These systems analyse patient data, medical records and symptoms to assist healthcare providers in making accurate diagnoses and treatment decisions.^8^ Furthermore, ML algorithms can analyse genetic, environmental and lifestyle factors to develop tailored treatment plans and health monitoring strategies for patients.^9^

Recent studies have highlighted the potential of AI, particularly ML, in neurosurgery—including tumour identification, prognostication of surgical outcomes and epilepsy management—thereby augmenting patient care through sophisticated and precise methodologies.^10^ Advances in robotic surgery research have also contributed to greater precision during surgical procedures, facilitated by the processing of real-time data. Integrating AI into surgical robots enables more precise control, which may lead to improved treatment outcomes and shorter patient recovery times.^11^

As technological progress continues, further advances in AI integration across healthcare, medical research and medical education are expected to revolutionise how medical professionals diagnose, treat and teach, ultimately improving patient outcomes and advancing medicine overall.^12^,^14^

However, a research disparity remains between the development of robust algorithms and the practical implementation of AI systems in healthcare.^15 16^ Despite the availability of powerful algorithms, their routine clinical use is still limited. Recent reviews have identified regulation, privacy, legal risk, ethics, clinical impact and cost as key barriers to adoption.^17^,^19^ These challenges underscore the complex issues that healthcare organisations and stakeholders must navigate to effectively integrate AI into clinical practice.

These findings highlight the need for further research in real-world clinical settings, emphasising that despite promising early developments, the clinical integration of AI technologies remains a work in progress.

The potential of AI to enhance medical practice and improve patient care is vast. However, numerous unanswered questions and unresolved issues remain. The widespread adoption of AI in medicine presents various challenges and ethical considerations that must be addressed to ensure the responsible and effective use of AI technologies.^20^,^22^

This has led to intense discussions about the opportunities and risks associated with this technological development. In the medical context, the development and implementation of AI-based technologies are crucial not only at a technological level but also at a social level. Ensuring the acceptance of such technologies by physicians and patients, for example, is essential for their successful integration and use in practice. This is particularly important given the highly sensitive nature of the data involved and the potential implications for health, privacy and accountability. Factors such as autonomy, trust, experience and transparency significantly influence the acceptance of AI systems in medical practice.^23^

The relevance of stakeholder involvement in the implementation of new healthcare technologies and practices is reflected in many implementation research frameworks and theories, as it helps to understand the needs, perspectives and concerns of those who will be affected by the technology being introduced. Hogg *et al* also identify a knowledge gap in considering the perspectives of stakeholders beyond healthcare professionals (HCPs) in research.^24 25^ While HCPs are well represented, the expectations, fears and hopes of other stakeholders, such as healthcare students (future medical professionals), patients, developers and regulators, have received limited attention. Different groups may have distinct concerns, expectations and priorities; overlooking them can hinder the multistakeholder coordination that AI integration requires.^25 26^

While existing studies confirm the promise of AI, they also call for ethical and fair implementation that addresses uncertainties regarding its operation and impact. Fazakarley *et al* concluded that UK HCPs generally perceive AI as beneficial for patient care and workflow efficiency, but face barriers such as concerns around data security, infrastructural disparities and the need for transparency and trust to support successful implementation.^27^ Čartolovni *et al* explore how physicians, patients and managers fear an erosion of the patient–doctor relationship if AI is poorly integrated, and Wubineh *et al* synthesise global evidence while noting that most empirical work continues to focus on expert users.^28 29^ Collectively, these studies highlight ongoing uncertainties about how AI will function in clinical settings and underscore the importance of capturing the full spectrum of stakeholder perspectives.^30 31^

This paper extends this line of work by investigating the opportunities and challenges associated with integrating AI in the healthcare sector, focusing on the perspectives of five stakeholder groups: lecturers, clinicians, healthcare students, AI experts and institutional stakeholders. It aims to fill the research gap by empirically analysing these diverse views on AI implementation in healthcare, enhancing understanding, addressing concerns, promoting acceptance and facilitating successful integration into medical practices.

## Methods

The study builds on a previous paper that examined stakeholder insights into AI competencies in medical education. It was conducted at the Medical Faculty and the Faculty of Science of the University of Tübingen as part of the project, ‘TüKITZMed—Tübingen KI-Trainingszentrum für die Medizin’ (Tübingen AI Training Center for Medicine) funded by the German Federal Ministry of Research, Technology and Space (BMFTR, funding number 16DHBKI086). In the present research, the same dataset is re-examined to explore an additional research question.^32^

### Participants and setting

The selection of participants followed a purposive sampling strategy, aimed at maximising heterogeneity to capture a wide range of perspectives relevant to the research question. Rather than selecting cases at random, participants were chosen based on their ability to provide rich, detailed information on the integration of AI in healthcare, ensuring content representativeness.^33^ The selection process was guided by the aim of including diverse perspectives critical to the integration of AI into medical education. The recruitment approach was tailored to the regional scope of the project and ensured the inclusion of local expertise, with a focus on stakeholders with significant experience in the field.

Stakeholders were categorised into five groups based on their primary professional role and expertise relevant to AI implementation in healthcare. Healthcare students included individuals studying human medicine, molecular medicine or medical technology (both clinical and preclinical stages), as well as doctoral candidates and students in their practical year. Lecturers were defined as university educators from either the medical faculty or the faculty of science who are involved in medical teaching or data science subjects relevant to AI, including computer science, mathematics and bioinformatics. Clinicians included practising physicians from various medical specialties, many of whom also served as educational coordinators or held academic teaching roles. AI experts were individuals with recognised technical expertise in ML, data science and bioinformatics, many holding senior academic or research positions in applied AI. Finally, institutional stakeholders were defined as staff members in academic, clinical or administrative leadership roles within the university or medical faculty, including those responsible for curriculum development, ethics and policy oversight, quality assurance or strategic planning in healthcare education and clinical delivery. In cases where individuals could be assigned to more than one category (eg, a clinician also leading an AI research group), categorisation was based on their primary role as reflected in the interview. An overview of the sample, including representative roles and demographic characteristics by group, is provided in table 1 in the results section.

**Table 1:** Stakeholder overview

Stakeholder group	n	Gender distribution	Age range	Representative roles
Healthcare students	10	5 female/5 male	19–28	Students from human medicine (preclinical, clinical, final-year interns, doctoral candidates), molecular medicine and medical technology.
Clinicians	9	1 female/8 male	37–58	(Hospital-based) physicians in neurosurgery, radiology, internal medicine including clinical coordinators and department heads.
Lecturers	6	1 female/5 male	38–42	University lecturers and academic researchers in medicine, computer science, mathematics, bioinformatics.
AI experts	6	2 female/4 male	35–50	AI researchers and data scientists with expertise in machine learning, biomedical data science, applied bioinformatics and health informatics; includes research group leaders and directors of academic and clinical data departments.
Institutional stakeholders	7	3 female/4 male	29–59	Staff in academic, clinical and administrative leadership roles responsible for curriculum development, ethics oversight, quality management and strategic planning in healthcare education and delivery.

AI, artificial intelligence.

Other stakeholder groups—for example, patients and political actors—were not included, as the focus was on the educational and professional aspects of AI integration rather than its impact on patient care or broader policy issues. Further details on the recruitment procedure and stakeholder classification have been published in a previous article based on the same dataset.^32^ To ensure transparency, the semistructured interview guide used in both studies is provided in the online supplemental appendix.

### Procedure and data collection

Data were collected through semistructured interviews conducted by the first author to explore different perspectives on the challenges and potential directions for AI implementation in healthcare. This qualitative approach allowed participants to express their views openly and fully, capturing a broad range of experiences. It reflects the early stage of research in this field, where the aim is to generate new hypotheses rather than test predefined ones. This approach was chosen to explore the various dimensions and perspectives of stakeholders involved in the integration of AI into medical education.

The total number of interviews was not predetermined; instead, data collection followed the principle of theoretical saturation, which is common in qualitative research.^34 35^ This means the topic was explored until no new insights were expected from further interviews. The semistructured questionnaire provided a guiding framework with predefined questions while allowing flexibility to respond to participants’ spontaneous input.

For this part of the study, the interviewer asked the following main questions, which specifically focused on participants’ views regarding the future development of AI in medicine:

- How do you see the impact of AI in medicine on your professional life in the future?
- In which areas of healthcare do you see challenges or potential for progress through the use of AI-related technologies?
- Where do you see the main challenges and opportunities in this development?

The recruitment period began on 5 August 2022 and continued until 31 March 2023, concluding with the final interview. Interviews were conducted either in person or via video conferencing. Each interview was audio recorded and subsequently transcribed verbatim for analytical purposes. All participants provided written informed consent prior to the interviews.

### Data analysis

The interview transcripts were analysed using a content structuring approach based on Kuckartz’s method.^36^ This involved both deductive and inductive category development. Deductive categories were initially derived from the semistructured interview guide, reflecting key research questions and theoretical assumptions and were used to guide the initial coding process. In parallel, inductive categories were developed from the data itself, capturing emergent themes and ideas that had not been anticipated.

Coding was conducted using MAXQDA 2022 (VERBI), with the flexibility to refine the category system as new insights emerged. To ensure reliability, intercoder agreement was established by having multiple researchers independently code a portion of the transcripts, followed by a comparison of results.^37^ Discrepancies were discussed and resolved collaboratively, leading to a refined and consistent coding scheme. This process enhanced the robustness of the analysis by reducing subjective bias.

## Results: opportunities and challenges of AI in medicine: a stakeholder’s view

To contextualise the perspectives presented in this study, table 1 provides an overview of the participants by stakeholder group, including demographic information and representative professional roles. The participants’ ages ranged from 19 to 59 years (N=38; M=38.5; SD=9.7; SEM=1.6), with two participants (one from the healthcare students’ group and one from the clinician’s group) not disclosing their age. The overall gender distribution was 26 male and 12 female participants.

**Figure 1:**
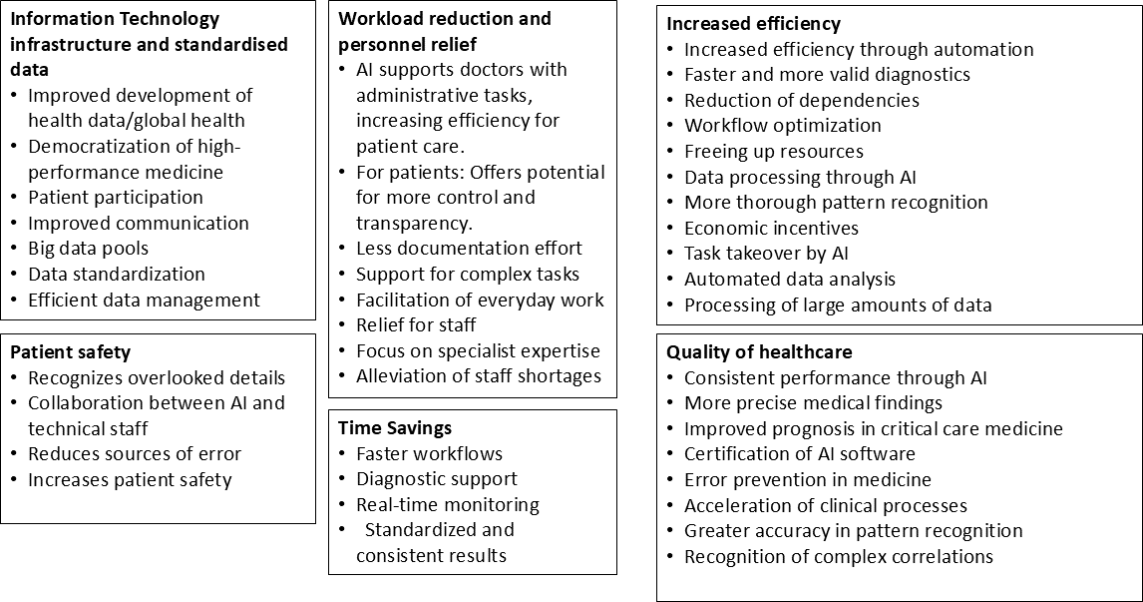
Opportunities for implementing AI in healthcare, as identified by stakeholders. AI, artificial intelligence.

Through qualitative content analysis, two main categories emerged (perceived challenges and limitations and perceived opportunities), comprising a total of 14 subcategories (see figures1  2).

**Figure 2:**
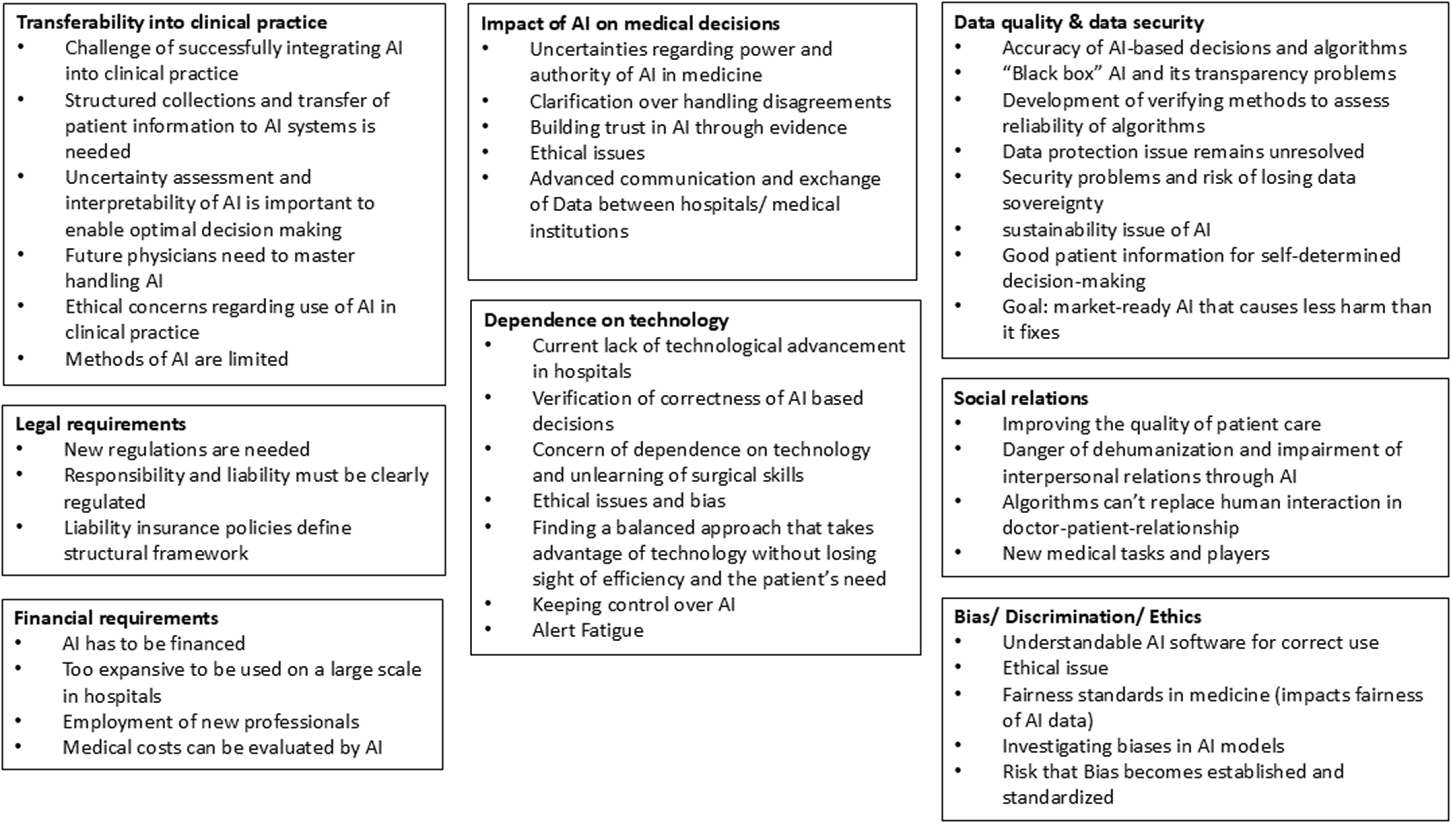
Challenges for implementing AI in healthcare, as identified by stakeholders. AI, artificial intelligence.

The results are organised according to the main and subcategories that emerged from the interviews. These categories were identified through an iterative, inductive–deductive coding process and reflect themes that recurred across interviews. Each theme is presented alongside the perspectives of all relevant stakeholder groups, allowing readers to see which aspects each group emphasised without imposing direct one-to-one comparisons. This structure captures the diversity of views while avoiding premature generalisation or polarisation. To enhance transparency regarding the distribution of perspectives, we compiled two tick matrices. Table 2 summarises which of the five stakeholder groups explicitly addressed each opportunity subcategory, while table 3 presents the same for the challenge subcategories. A tick in a given cell indicates that at least one interviewee from that group mentioned the corresponding theme.

**Table 2:** Stakeholder coverage of perceived opportunity subcategories (✓=subcategory mentioned)

Stakeholder group	IT infrastructure and standardised data	Increased efficiency	Workload reduction and personnel relief	Patient safety	Time savings	Quality of healthcare
Clinicians	✓	✓	✓	✓	✓	✓
Lecturers	✓	✓	✓		✓	
Healthcare students			✓	✓	✓	✓
AI experts	✓	✓	✓			✓
Institutional stakeholders	✓	✓		✓	✓	

AI, artificial intelligence; IT, Information Technology.

**Table 3:** Stakeholder coverage of perceived challenges subcategories (✓=subcategory mentioned)

Stakeholder group	Impact on medical decisions	Dependence on technology	Bias, discrimination, and ethics	Transferability into clinical practice	Social relations and doctor–patient communication	Data quality and security	Financial requirements	Legal requirements
Clinicians	✓	✓	✓	✓	✓	✓	✓	✓
lecturers	✓	✓	✓	✓	✓	✓	✓	✓
Healthcare students	✓	✓	✓	✓	✓	✓	✓	✓
AI experts		✓	✓	✓		✓	✓	✓
Institutional stakeholders	✓	✓	✓		✓	✓	✓	✓

AI, artificial intelligence.

### Perceived opportunities of AI in medicine

#### IT infrastructure and standardised data

Considering the various stakeholder groups, it is clear that establishing a solid IT (Information Technology) infrastructure and data standardisation is central to the medical landscape. AI experts emphasised that patient access to their own medical data and research results can increase participation and stimulate interest in clinical research. At the same time, this can help physicians and researchers access large datasets. Clinicians viewed AI as an important resource, particularly for developing countries with limited resources. They highlighted the potential to democratise medical care across locations, provided an internet connection is available. Data standardisation was seen as key to potentially improving the reproducibility of information.

Lecturers, in turn, emphasised that broad access to medical care across locations—provided there is a reliable internet connection—can help democratise high-performance medicine. They also noted the potential for increased reproducibility of data. Institutional stakeholders called for improved communication between hospitals and research institutions. They emphasised the creation of big data pools for specific diseases enabled by cross-site data management and collaboration. This opens new opportunities for research and helps reduce errors in physicians’ daily work. Increasing computing power and the use of data contribute to improving patient safety.

#### Increased efficiency

Clinicians emphasised that AI increases efficiency by organising and automating processes, such as infection diagnostics. This leads to faster, more accurate diagnoses and enables quicker surgeries with fewer complications. It also reduces dependence on individual clinicians, making medical practice more robust and allowing resources to be reallocated to avoid unnecessary tasks. The potential to use skilled personnel more effectively to address shortages and increased workload was also highlighted.

Lecturers acknowledged that while AI increases efficiency, it does not replace HCPs but supports them in managing large data sets, particularly in pattern recognition and similarity detection. They noted that AI can perform certain tasks more thoroughly in specific medical areas compared with humans. The economic incentives for using AI in medicine were also emphasised, especially in light of the physician shortage. Healthcare students did not comment specifically on this matter.

AI experts emphasised the increase in efficiency gained by assigning tasks that exceed human capabilities, where AI can process large volumes of data that would be difficult for humans to interpret. Institutional stakeholders noted the efficiency gains from automated data analysis and the evaluation of large data sets, which were previously labourious and time-consuming. They emphasised the opportunity to use extensive data collections more quickly and effectively.

#### Workload reduction and personnel relief

Clinicians emphasised that AI could support medical personnel by handling complex and time-consuming tasks, such as interpreting medical imaging scans. By automating these interpretations, AI helps to relieve overburdened nurses and physicians, allowing them to focus more on direct patient care and other critical responsibilities, improving overall efficiency and reducing workload stress. At the same time, they highlighted the vulnerability and dependence on these systems, underscoring the importance of reliable and robust AI functionality.

Lecturers saw AI as a helpful tool in day-to-day work, noting that computer-based analyses can lead to better results and ease the workload for staff. AI could also provide users with information that computers can process more efficiently.

Healthcare students highlighted AI’s potential to reduce and simplify their workload, for example, by serving as a memory aid to manage the large volume of information and tasks. They saw the automation and optimisation of workflows in medical facilities as a way to ease hospital overload. This, in turn, improves efficiency, frees up more time for patients and relieves pressure on medical staff.

AI experts emphasised that in practice, AI supports and relieves doctors, particularly with administrative tasks, leading to greater efficiency and more time for direct patient care. They also highlighted the potential for patients to gain more control, visibility and transparency. The ongoing development of digitisation was seen as crucial for the future, enabling workload reduction through less documentation and allowing doctors to focus on reviewing unusual findings and accelerating diagnoses.

#### Patient safety

Clinicians highlighted that AI can accelerate and simplify work processes and detect aspects that HCPs might overlook. At the same time, they emphasised the need for human experts to verify the accuracy of AI-generated results. Looking ahead, they expressed the desire for optimal collaboration between AI and human experts in diagnostics and treatment. Patients can place trust in AI-assisted examinations when they are reviewed and validated by HCPs. Clinicians also suggested that AI should be treated as a medical product and subject to formal certification. Healthcare students noted AI’s potential to enhance patient safety by reducing clinical errors. Institutional stakeholders underscored that reducing human error through the use of AI can significantly improve patient safety in routine care.

#### Time savings

Clinicians noted that rapid data processing can accelerate workflows involving many manual steps that are time-intensive and labour-intensive, such as radiation treatment planning. Automation through AI allows doctors to devote more time to meaningful activities such as patient interactions and care. Lecturers viewed AI as a support system that saves time and offers diagnostic assistance, although it was not considered a decisive factor. Healthcare students observed that AI enables faster surgical procedures through real-time monitoring, transmission and analysis. Institutional stakeholders emphasised the benefits of standardisation, which leads to more consistent results. This is achieved through rapid and continuous work, free from fatigue or distraction. Large volumes of data can be analysed swiftly and efficiently.

#### Quality of healthcare

Clinicians recognised that AI could provide consistent and reliable diagnostic performances regardless of external factors such as time of day or practitioner fatigue. It may also lead to more precise and accurate medical findings, especially in image-based assessments. Lecturers highlighted that software certification contributes to diagnostic reliability and standardisation. Healthcare students noted that AI can help reduce errors in medicine by supporting decision-making in situations where human limitations might otherwise lead to mistakes.

AI experts emphasised that AI systems can simultaneously analyse numerous data points, enabling faster and more accurate medical decisions. They stressed that this processing supports improved pattern recognition and the identification of complex clinical relationships—especially where human cognition reaches its limits.

### Perceived challenges of AI in medicine

Alongside the opportunities, stakeholders identified significant challenges in integrating AI into healthcare, highlighting the complexities and barriers that must be addressed for successful implementation (also see figure 2).

#### Impact of AI on medical decisions

Clinicians expressed mixed feelings about AI’s role in medicine. There was uncertainty about the power and authority AI should have in making medical decisions. Some were worried that AI might overshadow their expertise, stating that physicians need to maintain core skills and avoid over-reliance on AI. Ethical concerns, including patient privacy and data security, also remained significant.

Clinicians and healthcare students shared a common concern—the fear that overreliance on AI might lead to neglecting important basic technical skills. AI should be regarded as a valuable tool, not a replacement for fundamental medical knowledge and abilities. Lecturers faced the challenge of preparing future HCPs for a world where AI plays a crucial role in diagnostics and decision-making. They stressed the need for clear guidance on how to handle disagreements between physicians and algorithms in medical diagnostics. Institutional stakeholders highlighted the importance of advanced communication and data exchange between hospitals and medical institutions. They also emphasised that effective AI use requires performance evaluation through clinical benchmarking and comparative testing against human experts, providing a solid foundation for trust and integration.

#### Dependence on technology

All stakeholders expressed concerns about dependence on technology, particularly the fear that regular use could lead to a loss of surgical skills and the ability to accurately evaluate results. This concern was heightened by the possibility that if the technology fails or produces incorrect results, operations might be performed incorrectly. Clinicians noted the current lack of technological advancement in hospitals, which made them favour standardised applications and easily interpretable databases. They emphasised that verifying, monitoring and evaluating the accuracy of AI-based decisions requires an understanding of the underlying processes. Lecturers stressed the need to examine evidence for both medical and AI-assisted products, while considering ethical issues, individual autonomy, accessibility and equity, especially for populations with varying levels of health literacy. They emphasised the importance of balancing technology’s advantages with a continued focus on efficiency and patients’ needs. Additionally, they highlighted the medical profession’s role in making recommendations and explaining AI use to patients, taking into account public health measures and commercial interests. Excessive technologisation was seen as a potential drawback, possibly causing delays in patient transportation and ultimately disadvantaging patients.

Healthcare students focused on the importance of maintaining control over knowledge and AI-based decisions, expressing a genuine fear of the risks associated with self-optimisation and the lack of controllability of AI. AI experts cautioned that compulsive adaptation to technical solutions often led to frustration and negative outcomes, particularly when AI-based decisions were difficult to understand. At the institutional level, there was expressed concern that AI technologies could reduce the time spent with patients, interfere with autonomous decisions by patients and physicians, neglect the relational aspect of care, and fail to recognise patient individuality. Alert fatigue was identified as a problem preventing the effective prioritisation of warnings.

#### Bias, discrimination and ethics

Clinicians emphasised the importance of AI software being understandable for correct use. They stated that software must be designed so that users can accurately understand and evaluate information, as AI functioning as a ‘black box’ with unverifiable outputs raises ethical concerns, compromising users’ autonomy and responsibility.

Lecturers highlighted the need for fairness standards in medicine. They noted that studies have shown human physicians sometimes make poor clinical decisions when treating marginalised social groups, and similar problems could arise with AI. They raised the issue of discrimination, as AI may be trained on data from specific demographics, leading to bias in datasets. They also considered it important to have balanced datasets that take social criteria into account to avoid potential bias and unfair outcomes. Both lecturers and healthcare students stated that AI can only learn from existing data and cannot exceed that knowledge. They expressed concerns about AI training focusing on specific groups, potentially excluding other ethnic groups and minorities. Healthcare students also noted that the majority often benefits from data, while minorities are excluded. They identified ethical dilemmas, such as when AI calculates life expectancy for life-support decisions. Experts emphasised the importance of investigating biases in AI models, especially black box models. At the institutional level, it was acknowledged that the risk of biased AI systems becoming accepted as normal and standardised, even though they may not be equally suitable for all individuals. They noted that every algorithm and technique carries some risk of failure.

#### Transferability into clinical practice

Clinicians noted the challenge of developing daily applications that go beyond the research setting. They struggled to collect and transfer structured patient information to AI systems, particularly since personal and individual aspects are difficult to quantify. They emphasised that new physicians need to master the skill of understanding which tasks AI can perform.

Lecturers highlighted the importance of uncertainty assessment and AI interpretability to enable optimal decision-making. They stressed the need to demonstrate AI’s benefits, explore the human–AI interface and foster acceptance among medical professionals. They acknowledged the limitations of AI methods, particularly in recognising patterns and features that humans can detect. They also emphasised the need to equip future physicians and medical researchers with the skills to handle AI, noting that its rapid integration into daily practice is essential given its growing relevance.

Healthcare students expressed ethical concerns about AI in the context of palliative therapy and end-of-life care, particularly regarding decisions about switching off machines and ending life-prolonging measures. They emphasised the need to understand AI in order to use it effectively, noting that it remained a niche topic, mainly for those with a specific interest. Experts acknowledged the challenge of successfully integrating AI into clinical applications and turning it into a functioning product.

#### Social relations and doctor–patient communication

Clinicians expressed a desire to improve the quality of patient care by introducing AI that could give doctors more time to interact with their patients. Lecturers highlighted the potential dangers of dehumanisation and the weakening of interpersonal relationships resulting from the introduction of digital and technical applications in medicine. They also raised concerns about new players with commercial interests entering the healthcare system and the emergence of new medical tasks that might alter the doctor–patient relationship, potentially undermining trust. Healthcare students and institutional stakeholders recognised that algorithms cannot completely replace human interaction, emphasising the importance of the doctor–patient relationship in therapy. They stressed the need to strike a balance and avoid neglecting the human component by overusing AI applications.

#### Data quality and data security

Clinicians emphasised that AI performance depends on algorithm quality and the size and consistency of the underlying datasets. Small, non-standardised data pools and fragmented IT structures limit differential diagnosis, while ‘black-box’ models raise concerns about transparency and sound clinical judgement. They called for greater data availability and verifiable algorithm accuracy to support patient-centred decision-making. Lecturers noted that non-transparent algorithms hinder shared decision-making and that reliable, privacy-compliant access to large, high-quality datasets remains difficult. They stressed the need for methods to assess algorithm reliability, robust quality control to avoid bias, and clear physician responsibility for questioning AI outputs. Healthcare students emphasised the need for large, representative datasets and warned that small data pools may overlook rare diseases. They also pointed to unresolved data-protection issues, ongoing security risks and the danger of over-reliance on AI systems. AI experts highlighted the general scarcity of large, homogeneous datasets, the resource-intensive nature of data collection and the vulnerability of algorithms to data-protection gaps. They identified data availability—and the need to comply with data guidelines—as the main key to meaningful AI use. Institutional stakeholders recognised that AI systems require vast, standardised data resources yet may still reach their limits in individual cases. They underlined challenges in cross-site data exchange, patient-data governance and legal certainty, noting that data protection remains a central but unresolved issue. The possibility of software bugs leading to erroneous outputs further underscores the need for continuous verification.

#### Financial requirements

Clinicians emphasised that AI represents a financial issue, noting that its implementation and use carry cost implications. Lecturers suggested that in the future, AI could potentially be used to evaluate diagnoses, treatment outcomes and cost coverage. Healthcare students expressed concern that AI might be too expensive for large-scale use in hospitals, suggesting that financial constraints could limit its widespread adoption. Experts highlighted the broader financial implications, questioning who would be employed, or whether anyone would be employed at all, in the context of AI integration and its impact on the healthcare workforce.

#### Legal requirements

Clinicians emphasised the need for clear regulations, especially in cases where AI errors occur. They propose shifting responsibility from users to manufacturers and relaxing data protection rules for non-individually identifiable data.

Lecturers noted the underdeveloped legal landscape and certification processes for AI in the medical context, particularly in the absence of robust algorithms. Healthcare students raised concerns about liability, especially as AI takes on a larger role in patient care. Experts stressed the importance of defining liability laws and adapting legal frameworks in advance. At the institutional level, addressing legal requirements for AI in healthcare was recognised as crucial.

## Discussion

The survey of stakeholders revealed a wide spectrum of views regarding the perceived opportunities and challenges of implementing AI in healthcare. Opinions ranged from enthusiasm for the technology and positive assessments to scepticism and concern. By examining the collective discourse, we identified critical areas of concern, such as the need to establish a reliable IT infrastructure, enhance efficiency and reduce the workload of HCPs. This thematic analysis showed how different stakeholder groups—healthcare students, clinicians, AI experts, lecturers and institutional stakeholders—shared common interests and challenges, offering insights into their collaborative needs for effectively implementing AI in medical education and practice. This comprehensive understanding reflects the dynamic interplay between technology, professional roles and educational frameworks in shaping the future of healthcare. The inter-relatedness of different perspectives is essential for understanding how various groups influence and shape one another’s expectations and interpretations of AI. With regard to medical education, other authors have argued that it is increasingly shaped by broader technological and societal transformations, requiring a reconceptualisation of how knowledge, identity and power are negotiated within clinical training.^38^ For instance, while clinicians might emphasise efficiency and workload reduction, their views may be informed by the educational experiences of healthcare students or the technical insights of AI experts. By focusing on common themes, we can identify areas of agreement and potential conflicts that may not be apparent when analysing each group in isolation.

### Dimensions of perceptions of AI implementation in medicine

The results suggest that the concept of AI in medicine is associated with many hopes and expectations, particularly among stakeholders, with a medical background. These expectations include increased efficiency in clinical care, reduced workload for medical staff and more time for patients. However, concerns and perceived challenges remain, such as technologisation, potential biases in training data and unclear effects on the doctor–patient relationship. The extent to which opportunities and challenges were perceived varied depending on the context and background of the stakeholders surveyed.

The diversity of perspectives and viewpoints enables a thorough assessment of the potential impact of AI on medical care. It helps to clarify the interests, concerns and priorities of the stakeholders involved. This, in turn, allows decision-makers to make informed choices that not only enhance the effectiveness and efficiency of AI systems in healthcare but also increase patient and professional confidence in these technologies.^39^

The diversity and breadth of themes repeatedly mentioned by stakeholders underscore the presence of numerous expectations and concerns, aligning with findings from recent studies that explore the potential challenges and opportunities of AI implementation in healthcare.^3040^,^44^ In examining the perceived opportunities for integrating AI into healthcare, several recurring themes emerged across the subcategories (table 4). Central to these is the role of AI as a promising avenue to enhance various aspects of healthcare.

**Table 4:** Central derived themes from the subcategories related to opportunities for implementing AI

Efficiency improvement	Enhancing the efficiency of healthcare processes by automating tasks, optimising workflows and reallocating resources to streamline operations and boost productivity.
Quality enhancement	Improving the quality of healthcare service through more precise medical findings, improved diagnostics and error prevention, ultimately aiming for better patient care and outcomes through advanced technologies.
Data utilisation	Standardising, improving accessibility and ensuring interoperable data management to support informed decision-making and improved patient care.
Patient-centric approach	Using AI as a tool to empower patients, enhance communication and improve overall patient outcomes.
Technological infrastructure	Establishing a robust IT infrastructure, including the development of health data systems, democratisation of high-performance medicine, and support for AI implementation in healthcare.

AI, artificial intelligence.

The themes highlight the perceived benefits of integrating AI into healthcare, aim to drive positive change, increase efficiency and enhance quality in the healthcare sector through the strategic incorporation of AI technologies. However, stakeholders also identified challenges and risks, including uncertainties and ethical concerns related to AI’s role in medical decision-making, technical obstacles concerning infrastructure and safety management, and difficulties in seamlessly integrating AI into clinical workflows (table 5).

**Table 5:** Central derived themes from the subcategories related to challenges of implementing AI

Uncertainties and ethical issues	Uncertainties regarding the role of AI in medical decision-making processes, along with ethical dilemmas related to bias, fairness and trust in AI systems.
Technical challenges	Technical hurdles such as inadequate infrastructure in healthcare settings, and concerns about the accuracy, transparency, and security of AI-generated decisions.
Transferability to clinical practice	Difficulties in integrating AI into clinical workflows, including barriers to data collection and transfer, challenges in uncertainty management, limited interpretability of AI results and ethical considerations in clinical settings.

AI, artificial intelligence.

The assessment of the extent of perceived opportunities and challenges varied depending on the context and background of the surveyed actors. During the survey period, AI integration in healthcare was still in progress, and widespread implementation had not yet been achieved.^45^ Most stakeholders, except for AI experts with deeper insights into the subject, shared their views based primarily on the current state of AI development in medicine, drawing more on their perceptions and personal assessments than on actual experiences or real-world scenarios.

The study by Sartori and Bocca found that competence influences how people emotionally respond to various AI-related scenarios. Individuals with more technical knowledge tend to have a more positive attitude towards AI, while those with less competence are more likely to express concern. A lack of understanding or competence in AI may lead some to resist or refuse its adoption. Conversely, non-experts risk misusing AI or failing to leverage its full potential.^46 47^ It is essential to ensure that expectations and opinions are grounded in fact-based knowledge and personal experience, rather than speculation or hearsay, to avoid misinformation.^48^

Involving experts and non-experts in the AI implementation process is crucial for developing technologies that are not only technically robust but also socially responsible, inclusive and aligned with the values and needs of broader society. The hopes and fears surrounding AI significantly shape societal perceptions and arrangements of technology, especially in fields like medicine, where multiple stakeholders, including patients, either directly use AI or are affected by its implementation.^47^ While such concerns and expectations have been described in earlier studies, our analysis adds a systematic comparison of five stakeholder groups within a single dataset. This approach allows us to identify both shared hopes—such as improved efficiency or quality of care—and role-specific concerns, particularly among healthcare students and educators, who emphasised transparency, trust and digital dependency.^27 29^ By including both expert and non-expert voices, our study reveals how institutional role shapes interpretations of AI and highlights the importance of aligning these diverse expectations for successful implementation.

### Understanding the dynamics of AI implementation in healthcare

AI undeniably holds enormous potential to revolutionise various areas of medicine. However, realising this potential requires the use of reliable, functional and above all, safe AI technologies that prioritise patient safety and well-being.^49^ It is equally important to establish ethical principles, ensure data integrity and clarify legal frameworks.^50^ These elements form the foundation for a coherent understanding of the AI’s role and appropriate use in healthcare and have the potential to be embedded in future norms and standards. Such standards can support interoperability and promote collaboration between AI technologies and healthcare stakeholders. However, the perspectives of individual stakeholders—their awareness, knowledge and emotional responses to AI—cannot easily be incorporated into these norms and standards.^51^ Recognising and addressing these subjective elements is crucial for the effective integration of AI into healthcare. Narratives about AI not only reflect societal perceptions but also actively shape how the technology is understood, accepted and embedded into different areas of society.^52^

### AI in healthcare: hope, reality and the social construction of technology

As a general term, AI encompasses a range of technologies that evoke significant hopes and expectations. At the same time, many concerning visions exist regarding how AI might develop and affect society. Perceptions of AI’s capabilities fluctuate between technological promise and market-ready systems.^53^ The transfer of these technologies into real world applications remains pending and requires a suitable infrastructure for reliable use in medicine.^54^ While expectations for AI are high, the reality often lags behind.^47^ Currently, there is no all-encompassing AI system; instead, various individual technologies exist with specific, predefined functions. Legally, economically and ethically, the medical profession remains far from deploying self-adapting AI systems or a broadly usable key technology.^8 11 20^

A useful theoretical framework for understanding these dynamics is the social construction of technology (SCOT) model.^55^ This model emphasises that it is not technical necessities but rather the perceptions, views, strategies and practices of the actors directly or indirectly involved in the development process that determine the shape and functionality of a new technology. From this perspective, the implementation of AI in healthcare cannot be reduced to a simple technology push, in which powerful algorithms are developed in a lab and then rolled out to passive users.

**Figure 3:**
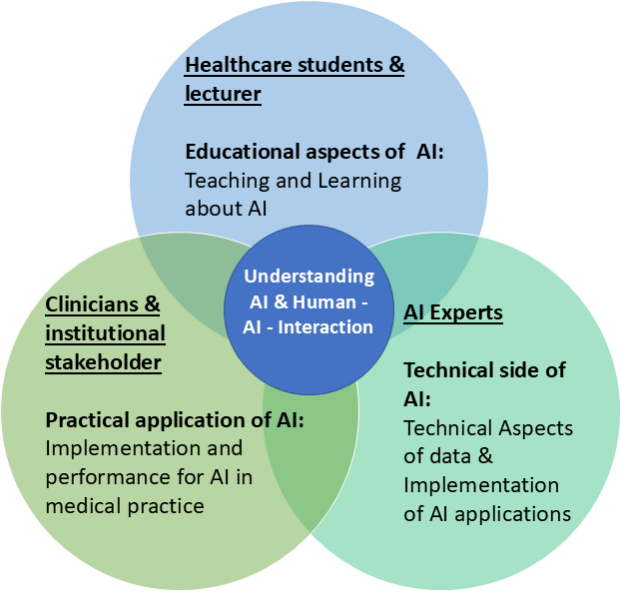
Inter-related sociotechnical aspects of integrating AI into medical practice: understanding stakeholders’ perspectives and needs regarding AI in medicine. AI, artificial intelligence.

The SCOT theory emphasises ‘interpretive flexibility’, indicating that various social groups may have different interpretations and expectations of a technology.^56^ Hopes and concerns circulate both within and across these groups. Whether these will ultimately materialise remains unclear, but they are already shaping attitudes that can either accelerate or derail implementation. By clustering the heterogeneous perceptions of different stakeholder groups into three intersecting domains (see figure 3), we argue that AI adoption is a continuously negotiated process among social groups whose priorities may or may not overlap. The areas of overlap indicate where partial consensus is emerging (eg, the need for transparent human–AI interaction), while the non-overlapping areas highlight issues that still require alignment. Finally, the diagram provides a snapshot of an ongoing social process.

This study suggests that successful AI deployment is not so much a ‘technology push’ but rather a multiactor negotiation. An inclusive approach that incorporates the perspectives of all relevant stakeholders can promote the stabilisation and establishment of AI technology in medical practice.^57^ To avoid resistance and unrealistic expectations, implementers must (1) engage these groups early and openly, (2) communicate clearly what current AI can and cannot do and (3) build AI literacy into medical training. Inclusive, expectation-managed roll-outs are essential for stabilising the technology in routine practice.

AI refers to many distinct tools, each raising high hopes and serious concerns. Because these tools will be used by clinicians, patients and other end-users, their limitations must be clearly stated. Meaningful public and professional dialogue—beginning before systems are built—helps align AI with the way people want to live and work. Early AI literacy in medical curricula, coupled with continuous user training, is therefore essential for maintaining ethical and professional standards as this disruptive technology matures.^58 59^

### Strengths and limitations

Although the qualitative approach of our study enabled an in-depth exploration and yielded valuable insights into stakeholder perceptions, the limited sample size may not adequately represent the broader population, potentially constraining the generalisability of the findings. Furthermore, the demographic composition of participants may not fully reflect the diversity of perspectives and experiences across different societal groups. As AI technologies and public awareness continue to evolve rapidly, some of the study’s findings may soon become outdated. Nevertheless, the study offers important initial insights that can inform future research and policy developments. Continued research will be necessary to keep pace with technological advances and shifting societal attitudes. Addressing these limitations in future studies will be key to developing a more comprehensive understanding of AI’s impact on healthcare.

## Conclusions

This study explored perceptions of AI development in medicine. The use of a semistructured interview guide allowed stakeholders to express their views freely, enabling a broad and nuanced understanding of individual perspectives. Our findings reveal shared hopes—such as improved efficiency, data standardisation and enhanced patient care—as well as group-specific concerns, including uncertainty, technical dependency and ethical or regulatory challenges. By examining participants’ levels of awareness, knowledge and emotional responses to AI, the study offers valuable insight into how different groups understand and engage with these technologies.

Importantly, these highlight the need to include non-expert perspectives in discussions about AI, as they raise distinct concerns that are vital for socially responsive and context-sensitive implementation. Aligned with a social constructivist view of technology, our results suggest that AI adoption is not just a technical process but a socially negotiated one. The concept of interpretative flexibility shows that stakeholders ascribe different meanings and priorities to AI depending on their roles and expectations.^60^ Recognising this key to avoiding resistance and ensuring that AI systems are meaningfully integrated into clinical and educational practice.

Building on the findings of our previous publication using the same dataset, which focused on identifying the AI competencies stakeholders consider essential for medical education, this study adds a complementary perspective by exploring the conditions under which AI implementation is perceived as feasible or problematic.^32^ The results offer practical guidance for translating stakeholder concerns and expectations into action, for example, by tailoring curricula to the needs of specific professional roles, involving non-expert users early in implementation processes and addressing institutional infrastructure. The study suggests that a participatory approach, involving stakeholders directly in the implementation process and considering their expertise, desires and concerns, is essential for the success of AI integration. While AI offers many opportunities, uncertainties about its reliability, validation and interpretability remain. Thus, transparency, explainability and patient involvement are vital for building trust in AI and alleviating concerns. Further research is needed to understand the criteria and mechanisms for trust in AI systems to ensure their successful integration into medical practice.

## Supplementary material

10.1136/bmjopen-2024-096208 online supplemental file 1

## Data Availability

Data are available on reasonable request.
