# Supplementary material for: Exploring the social dimensions of AI integration in healthcare: a qualitative study of stakeholder views on challenges and opportunities
Source: BMJ Open. 2025 Jun 27;15(6):e096208. doi: 10.1136/bmjopen-2024-096208 (PMC12207147; doi:10.1136/bmjopen-2024-096208)
Supplement: Supplementary file 2 [file bmjopen-2024-096208.reviewer_comments.pdf]

PEER REVIEW HISTORY

BMJ Open publishes all reviews undertaken for accepted manuscripts. Reviewers are asked to complete a checklist review form and are provided with free text boxes to elaborate on their assessment. These free text comments are reproduced below.

ARTICLE DETAILS

Title (Provisional)

Exploring the Social Dimensions of AI Integration in Healthcare: A Qualitative Study of Stakeholder Views on Challenges and Opportunities

Authors

Moldt, Julia-Astrid; Festl-Wietek, Teresa; Fuhl, Wolfgang; Zabel, Susanne; Claassen, Manfred; Wagner, Samuel; Nieselt, Kay; Herrmann-Werner, Anne

---

VERSION 1 - REVIEW

Reviewer 1

Name Paprica, P. Alison

Affiliation University of Toronto, Institute for Health Policy, Management and Evaluation

Date 01-Feb-2025

COI None

Overall, the manuscript presents results that are likely to be of interest to BMJOpen readers; however, more work needs to be done to (i) present the findings of the study in the context of previous work, (ii) help the reader understand the authors' interpretation of results, including by modifying Figures to identify which stakeholder groups expressed which views and (iii) focus the introduction and conclusions on the findings of the study (as opposed to the method used to generate findings).

The abstract should include information about what themes were identified. The wording in the abstract related to the primary outcome measure is not consistent with what a primary outcome measure is. Insufficient support is provided for the following text identified with CAPITALS in the conclusions of the abstract "Understanding these viewpoints can inform decision-makers, LEADING TO IMPROVED EFFECTIVENESS AND EFFICIENCY OF AI SYSTEMS in healthcare while FOSTERING CONFIDENCE AMONG PATIENTS and professionals.

The literature cited in the introduction should be expanded to include more studies and review articles that focus on stakeholder views about healthcare AI.

The authors should consider changing text in the manuscript that reads as subjective, e.g., “students, whose AI competencies are vital for both current education and future practice” could be written as “medical students.”

Ideally, the authors will provide their interview guide as supplementary material and include information about how they determined which of the stakeholder groups an interviewee belonged in (including information about what characteristics led to someone being identified as an AI expert and whether any participants belonged in more than one stakeholder category).

As written, the text in Section 3.2 is hard to process. Some of the subheadings of section 3.2 only include the views of one or two of the stakeholder groups. The authors may want to consider a large table that provides examples of the views of different stakeholder groups for each of the themes they identified. It seems that one of the authors’ main conclusions is that different stakeholder groups identified different themes, but Figures 1 and 2 do not present information about which stakeholder groups identified which themes. Additionally, the manuscript would be strengthened if the authors include illustrative examples of verbatim statements related to the main themes they identified. A large table, or other alternative formats, could help readers understand how the authors developed Figure 3 from their findings.

Figure 3 is probably intended to read “Students and Lecturers” not “Students and Lectures”

In their discussion section, the authors should identify which themes identified by their study are new or different from themes identified in previous studies.

The text in the conclusions section focuses mostly on the authors’ positive assessment of their method as opposed to what their study found. The authors should revise or provide a reference to support the claim “The findings emphasize the importance of including non-expert perspectives in AI discussions. Such viewpoints, often underestimated, are crucial for a comprehensive understanding of societal attitudes towards AI.”

As a minor issue, there are cases where there is variation in the how the in-text citations are presented (e.g. lines APA, vs. consecutive end notes, including but not limited to lines 99 and 129).

**Reviewer**	**2**
**Name**	**Patel, Ankush**
**Affiliation**	**Mayo Clinic**
**Date**	**05-Apr-2025**
**COI**	**None**

The authors provide valuable insight on the perspectives of AI integration into healthcare, with application of a theoretical framework apt for interpretation of findings. The manuscript may be revised for minor grammatical and language issues. Practical implications may be strengthened with specific recommendations. For example: an implementation framework or staged approach to AI integration in clinical settings with checkpoints for evaluation, educational recommendations such as curriculum components of medical education that enhance AI literacy, stakeholder engagement strategies, etc.

Some areas of the results section may also be condense to improve readability. Quality of healthcare and patient safety sections contain some repetitive elements concerning error reduction and reliability. Data security and errors may be streamlined during discussion of technical challenges. Some of the detailed stakeholder perspectives may be organized into comparison tables rather than sequential text paragraphs to mitigate repetition while highlighting similarities and differences effectively.

Overall an excellent work.

VERSION 1 - AUTHOR RESPONSE

Reviewer: 1

Dr. P. Alison Paprica, University of Toronto

Comments to the Author:

Overall, the manuscript presents results that are likely to be of interest to BMJOpen readers; however, more work needs to be done to (i) present the findings of the study in the context of previous work, (ii) help the reader understand the authors' interpretation of results, including by modifying Figures to identify which stakeholder groups expressed which views and (iii) focus the introduction and conclusions on the findings of the study (as opposed to the method used to generate findings).

Thank you for your general appreciation and the opportunity to improve our manuscript further. Please see our responses to the more specific comments below.

The abstract should include information about what themes were identified. The wording in the abstract related to the primary outcome measure is not consistent with what a primary outcome measure is.

We thank the reviewer for this helpful comment. In response, we revised the abstract to explicitly name the two main thematic categories identified in our analysis, along with illustrative subthemes. We also removed the term "primary outcome measure," as we agree that it is not appropriate in the context of a qualitative study.

We acknowledge that the phrase “primary outcome measure” may suggest a quantitative design. In accordance with the BMJ Open structured abstract requirements, we have kept the “Outcome Measures” heading but revised the wording to reflect the qualitative nature of the study. The

updated sentence avoids quantitative terminology and more accurately describes the thematic insights generated through content analysis.

Insufficient support is provided for the following text identified with CAPITALS in the conclusions of the abstract “Understanding these viewpoints can inform decision-makers, LEADING TO IMPROVED EFFECTIVENESS AND EFFICIENCY OF AI SYSTEMS in healthcare while FOSTERING CONFIDENCE AMONG PATIENTS and professionals.

Thank you for your comment. Furthermore, we rephrased the concluding sentence to more cautiously reflect the implications of our findings, avoiding overstatements and aligning more closely with the qualitative nature of the study as follows: “Diverse stakeholder perspectives provide valuable insights into the anticipated effects of AI in healthcare. Understanding these perspectives can support decision-makers in designing context-sensitive AI strategies and identifying areas for further professional and institutional development. Future research should monitor how these attitudes evolve in response to technological progress and real-world implementation.”

The literature cited in the introduction should be expanded to include more studies and review articles that focus on stakeholder views about healthcare AI.

Thank you for this helpful suggestion. Please keep in mind that this area of research is rapidly growing, and even the short time span between initial submission and the time of review might make a difference of several new papers on the topic. However, we do agree with your statement and have thus widened the introductory literature review to update and better reflect the rapidly growing body of work on stakeholder perspectives in the introduction section (lines 124-130). We added three recent multi stakeholder studies—Fazakarley et al. 2023, Čartolovni et al. 2023 and Wubineh et al. 2024, to illustrate current knowledge and remaining gaps.

The authors should consider changing text in the manuscript that reads as subjective, e.g., “students, whose AI competencies are vital for both current education and future practice” could be written as “medical students.”

Thank you for the suggestion. We have revised the wording accordingly; the manuscript now refers to this group as “medical students as future users of AI systems in clinical practice.” (p. 6).

Ideally, the authors will provide their interview guide as supplementary material and include information about how they determined which of the stakeholder groups an interviewee belonged in (including information about what characteristics led to someone being identified as an AI expert and whether any participants belonged in more than one stakeholder category).

Thank you very much for this helpful comment. As the current manuscript draws on the same interview data set as our previously published study (JMIR Med Educ 2024; doi: 10.2196/58355), we have already provided detailed information on participant recruitment and stakeholder classification in that paper. To avoid unnecessary duplication, we refer readers to this publication for the rationale behind group assignment and inclusion criteria. To enhance transparency in the current submission, we have additionally uploaded the semi-structured interview guide as supplement 1, as requested.

We hope this strikes a balance between transparency and conciseness while addressing your suggestion.

As written, the text in Section 3.2 is hard to process. Some of the subheadings of section 3.2 only include the views of one or two of the stakeholder groups. The authors may want to consider a large table that provides examples of the views of different stakeholder groups for each of the themes they identified. It seems that one of the authors' main conclusions is that different stakeholder groups identified different themes, but Figures 1 and 2 do not present information about which stakeholder groups identified which themes. Additionally, the manuscript would be strengthened if the authors include illustrative examples of verbatim statements related to the main themes they identified. A large table, or other alternative formats, could help readers understand how the authors developed Figure 3 from their findings.

We thank the reviewer for this valuable and constructive feedback. We acknowledge that the original presentation of section 3.2 was text-heavy and, in parts, challenging to follow. We agree that a more structured and accessible format can help readers better understand the perspectives of different stakeholder groups on the identified challenges. In response to your helpful suggestion, we carefully reviewed the overlapping subcategories related to data and reliability concerns. As a result, we decided to consolidate the following three subthemes: Security management and AI as a source of error, Quality of data and data sets, and Data security. These categories were closely connected conceptually, each addressing different aspects of what constitutes trustworthy and safe AI implementation in healthcare. We have now merged them into a single, integrated subcategory titled "Data quality & data security" (page 18). Accordingly, we have updated Fig. 2. We believe this restructuring not only streamlines the presentation but also improves clarity.

We appreciate the suggestion to illustrate the themes with verbatim excerpts. After careful consideration, however, we decided not to embed direct quotations in the manuscript. Including representative quotes for nine stakeholder–theme combinations would substantially lengthen Sections 3.1 and 3.2 and risk fragmenting the flow. A condensed thematic synthesis, supported by the tick-matrices, keeps the results section focused and easier to follow. Further, the study's objective is to compare patterns across groups rather than to showcase individual voices.

To compensate for the absence of verbatim statements, we created two overview tables (Table 1 and Table 2) that map which stakeholder groups addressed each of the identified opportunity and challenge subcategories. A tick indicates that at least one interviewee from a given group explicitly mentioned the respective theme. These matrices were added for a clearer representation of who expressed which views, particularly given that not all subthemes were addressed equally by all stakeholder groups. At the same time, we hope the tables offer a helpful bridge to Figure 3 (Venn diagram), as they show the empirical basis for the domain clustering (e.g., practical, technical-systemic, and educational aspects).

We hope that this integrated presentation better supports the reader's understanding and fully addresses your concern.

Figure 3 is probably intended to read "Students and Lecturers" not "Students and Lectures"

Thank you for catching that typo. We have corrected the label in Figure 3 to "Students and Lecturers".

In their discussion section, the authors should identify which themes identified by their study are new or different from themes identified in previous studies.

Thank you for this helpful comment. In response, we have revised the discussion section to clarify what is new and distinctive about our findings in comparison to previous stakeholder-focused studies on AI in healthcare. (Please see p. 25 and 26).

The text in the conclusions section focuses mostly on the authors' positive assessment of their method as opposed to what their study found. The authors should revise or provide a reference to support the claim "The findings emphasize the importance of including non-expert perspectives in AI discussions. Such viewpoints, often underestimated, are crucial for a comprehensive understanding of societal attitudes towards AI."

Thank you for this important suggestion. We have revised the conclusion section to shift the focus to a more substantive summary of our findings and included better support to the claim regarding the value of non-expert perspectives in AI discussions.

As a minor issue, there are cases where there is variation in the how the in-text citations are presented (e.g. lines APA, vs. consecutive end notes, including but not limited to lines 99 and 129).

Thank you for your valuable comment. We have revised the manuscript to ensure consistency in citation formatting throughout the text, aligning all references with the journal's required style.

Reviewer: 2

Dr. Ankush Patel, Mayo Clinic

Comments to the Author:

The authors provide valuable insight on the perspectives of AI integration into healthcare, with application of a theoretical framework apt for interpretation of findings.

We thank the reviewer for the positive feedback and the constructive suggestions. In response, we made several revisions to improve clarity and structure.

The manuscript may be revised for minor grammatical and language issues.

Thank you for this important suggestion. We have carefully revised the manuscript to address minor language inconsistencies and improve stylistic clarity throughout.

Practical implications may be strengthened with specific recommendations. For example: an implementation framework or staged approach to AI integration in clinical settings with checkpoints for evaluation, educational recommendations such as curriculum components of medical education that enhance AI literacy, stakeholder engagement strategies, etc.

Thank you for this valuable comment. We agree that structured implementation models—such as staged approaches or formal frameworks—can be valuable tools for guiding clinical and educational change. However, given the exploratory and qualitative nature of our study, our aim was not to prescribe fixed implementation pathways, but rather to uncover the underlying perceptions, expectations, and concerns that shape stakeholder attitudes toward AI integration. At this stage, our findings provide a foundation for future work that may build and test more formalized frameworks. In response, we have strengthened the discussion and conclusion by adding concrete references to curriculum development and institutional implementation strategies. In addition, we now clarify how this study complements our previously published findings on AI competencies in medical education by highlighting contextual conditions for implementation as perceived by diverse stakeholder groups. We hope that these clarifications meet your expectations and are satisfactory in addressing your valuable suggestions.

Some areas of the results section may also be condense to improve readability. Quality of healthcare and patient safety sections contain some repetitive elements concerning error reduction and reliability.

We thank the reviewer for this helpful observation. To improve clarity and reduce redundancy, we revised the Patient safety and Quality of healthcare sections to better differentiate their thematic focus. We retained all stakeholder perspectives but removed overlapping formulations regarding error prevention and reliability. We hope these revisions improve the readability of the section.

Data security and errors may be streamlined during discussion of technical challenges. Some of the detailed stakeholder perspectives may be organized into comparison tables rather than sequential text paragraphs to mitigate repetition while highlighting similarities and differences effectively.

We appreciate this helpful suggestion. In response, we streamlined and consolidated thematically related subcategories—such as Security management, Data quality, and Data security—into a unified theme (Quality of data and data security) to reduce fragmentation and repetition in the text (p. 19).

In addition, to improve accessibility and highlight group-specific emphasis across themes, we included two comparison tables (Tables 1 and 2, p.9) that map stakeholder groups to the subcategories of perceived opportunities and challenges.

Overall an excellent work.

Thank you for your general appreciation and your acknowledgment of the importance of our findings.

Reviewer: 1

Competing interests of Reviewer: None

Reviewer: 2

Competing interests of Reviewer: No competing interests.

VERSION 2 - REVIEW

Reviewer 1

Name Paprica, P. Alison

Affiliation University of Toronto, Institute for Health Policy, Management and Evaluation

Date 26-May-2025

COI

The authors' revisions have improved the manuscript significantly, but there are still some outstanding issues.

Foremost, the uploaded version (Manuscript_revised_tracked_final.docx) has many small errors (e.g., line 86 "These findings highlight the the need"), making me wonder if the wrong version was uploaded by mistake.

Most importantly, it is unclear who the authors interviewed. For example, I do not know what the authors mean by "institutional stakeholders" who are "involved in implementing AI curricula" – perhaps if they included some of the job titles of institutional stakeholders, that would clarify things. Similarly, I do not know what constitutes an AI expert according to the authors. I also note that the authors mostly use the term "medical students" but also use the term "healthcare students" or simply "students." It would help if the authors used the same term to refer to students throughout the manuscript and clarified whether "medical students" refers to people who are training to be MDs (medical doctors) or also includes "pre-med" or other healthcare students (e.g., nursing students).

Additionally, the authors should include more detailed information about the people they interviewed. Given that the manuscript focuses on presenting stakeholder views, I do not think it is appropriate to expect readers to go to a different article to access information about who the stakeholders were; I also note that there is no table with participant details in the article they suggested I consult (https://mededu.jmir.org/2024/1/e58355/).

In the absence of detailed information about participant characteristics (e.g., a table that presents one row for each stakeholder group alongside the ages, genders and (possibly) representative job titles) the information that the authors do provide about participants' ages and genders does not have a practical meaning (e.g., I cannot tell if there were any women AI experts).

Further, the text "The participants' ages ranged from 19 to 59 years(N = 36; M = 38.5; SD = 9.7; SEM = 1.6; 2 missing) , with a gender distribution of 26 males and 12 females" is confusing. The text should be revised to state n=38, and a detailed table as would be added

as a better way to present the ages and genders of the participants in each stakeholder group.

VERSION 2 - AUTHOR RESPONSE

Reviewer: 1
Dr. P. Alison Paprica, University of Toronto
Comments to the Author:
The authors' revisions have improved the manuscript significantly, but there are still some outstanding issues.

We sincerely thank the reviewer for the thoughtful feedback and for acknowledging the improvements made in the revised manuscript. We appreciate the opportunity to address the remaining issues and have carefully revised the manuscript to resolve the outstanding points.

Foremost, the uploaded version (Manuscript_revised_tracked_final.docx) has many small errors (e.g., line 86 "These findings highlight the the need"), making me wonder if the wrong version was uploaded by mistake.
Thank you very much for pointing this out. The issue was due to the use of Microsoft Word's "track changes" functions, which unfortunately masked some formatting inconsistencies, such as duplicated spaces, that remained in the tracked version but were not visible during our final review. We apologize for this oversight and have carefully proofread the manuscript again to ensure that all such minor errors have now been corrected.

Most importantly, it is unclear who the authors interviewed. For example, I do not know what the authors mean by "institutional stakeholders" who are "involved in implementing AI curricula" – perhaps if they included some of the job titles of institutional stakeholders, that would clarify things. Similarly, I do not know what constitutes an AI expert according to the authors. I also note that the authors mostly use the term "medical students" but also use the term "healthcare students" or simply "students." It would help if the authors used the same term to refer to students throughout the manuscript and clarified whether "medical students" refers to people who are training to be MDs (medical doctors) or also includes "pre-med" or other healthcare students (e.g., nursing students).

Thank you for this important thought. In response, we revised the *Methods section* (p.5) to include clearer definitions of all five stakeholder categories and specified the inclusion criteria for each group. For example, we now clarify that "institutional stakeholders" refers to staff in academic, clinical, and administrative leadership roles responsible for curriculum development, ethics, quality management, and strategic planning within university and clinical settings. Similarly, "AI experts" are described as individuals with advanced technical expertise in machine learning, data science, or bioinformatics, often holding senior academic or research positions.

In addition, we have added a comprehensive participant table in the *Results* section (Table 1, p.8), which includes representative roles and demographics by stakeholder group to further increase transparency.

To address the inconsistency in terminology, we have replaced all instances of “medical students” and “students” with the uniform term “healthcare students” throughout the manuscript. We also specify that this group includes students of human medicine, molecular medicine, and medical technology (including preclinical, clinical, and practical year stages).

Additionally, the authors should include more detailed information about the people they interviewed. Given that the manuscript focuses on presenting stakeholder views, I do not think it is appropriate to expect readers to go to a different article to access information about who the stakeholders were; I also note that there is no table with participant details in the article they suggested I consult (https://mededu.jmir.org/2024/1/e58355/).
In the absence of detailed information about participant characteristics (e.g., a table that presents one row for each stakeholder group alongside the ages, genders and (possibly) representative job titles) the information that the authors do provide about participants' ages and genders does not have a practical meaning (e.g., I cannot tell if there were any women AI experts).
Thank you for your comment. We fully agree that a more detailed overview of the participant characteristics strengthens the transparency and interpretability of our findings. In response, we have included a new summary table in the Results section (Table 1, p. 8), which presents the number of participants by stakeholder group along with their age range, gender distribution, and representative roles or job titles. We hope these additions address your concerns and make the stakeholder profiles more accessible and informative to readers.

Further, the text "The participants' ages ranged from 19 to 59 years(N = 36; M = 38.5; SD = 9.7; SEM = 1.6; 2 missing) , with a gender distribution of 26 males and 12 females" is confusing. The text should be revised to state n=38, and a detailed table as would be added as a better way to present the ages and genders of the participants in each stakeholder group.

Thank you for noting this inconsistency. The value “N = 36” was a typographical error and has been corrected to “N = 38.” As already described, we have added a detailed participant overview table (Table 1), which includes the number of participants per stakeholder group, their gender distribution, age ranges, and representative roles. We hope this clarifies the demographic composition of our sample as requested.

Reviewer: 1
If you have selected ‘Yes’ above, please provide details of any competing interests.: Not applicable

Thank you for your general appreciation and the opportunity to improve our manuscript further.

Reviewer: 1
If you have selected ‘Yes’ above, please provide details of any competing interests.: Not applicable
